# Cysteine Specific Targeting of the Functionally Distinct Peroxiredoxin and Glutaredoxin Proteins by the Investigational Disulfide BNP7787

**DOI:** 10.3390/molecules20034928

**Published:** 2015-03-18

**Authors:** Aulma R. Parker, Pavankumar N. Petluru, Vicki L. Nienaber, John Badger, Betsy D. Leverett, Kamwing Jair, Vandana Sridhar, Cheyenne Logan, Philippe Y. Ayala, Harry Kochat, Frederick H. Hausheer

**Affiliations:** 1 BioNumerik Pharmaceuticals, Inc., 8122 Datapoint Drive, Ste. 1250, San Antonio, TX 78229, USA; 2 Zenobia Therapeutics, Inc., 505 Coast Blvd. South, Suite 111, La Jolla, CA 92037, USA

**Keywords:** BNP7787, glutaredoxin, lung adenocarcinoma, non-small cell lung cancer, peroxiredoxin, sulfiredoxin, Tavocept^®^, thiol-disulfide exchange, thioredoxin

## Abstract

Glutaredoxin (Grx), peroxiredoxin (Prx), and thioredoxin (Trx) are redoxin family proteins that catalyze different types of chemical reactions that impact cell growth and survival through functionally distinct intracellular pathways. Much research is focused on understanding the roles of these redoxin proteins in the development and/or progression of human diseases. Grx and Prx are overexpressed in human cancers, including human lung cancers. BNP7787 is a novel investigational agent that has been evaluated in previous clinical studies, including non-small cell lung cancer (NSCLC) studies. Herein, data from activity assays, mass spectrometry analyses, and X-ray crystallographic studies indicate that BNP7787 forms mixed disulfides with select cysteine residues on Grx and Prx and modulates their function. Studies of interactions between BNP7787 and Trx have been conducted and reported separately. Despite the fact that Trx, Grx, and Prx are functionally distinct proteins that impact oxidative stress, cell proliferation and disease processes through different intracellular pathways, BNP7787 can modify each protein and appears to modulate function through mechanisms that are unique to each target protein. Tumor cells are often genomically heterogeneous containing subpopulations of cancer cells that often express different tumor-promoting proteins or that have multiple dysregulated signaling pathways modulating cell proliferation and drug resistance. A multi-targeted agent that simultaneously modulates activity of proteins important in mediating cell proliferation by functionally distinct intracellular pathways could have many potentially useful therapeutic applications.

## 1. Introduction

**Figure 1:**
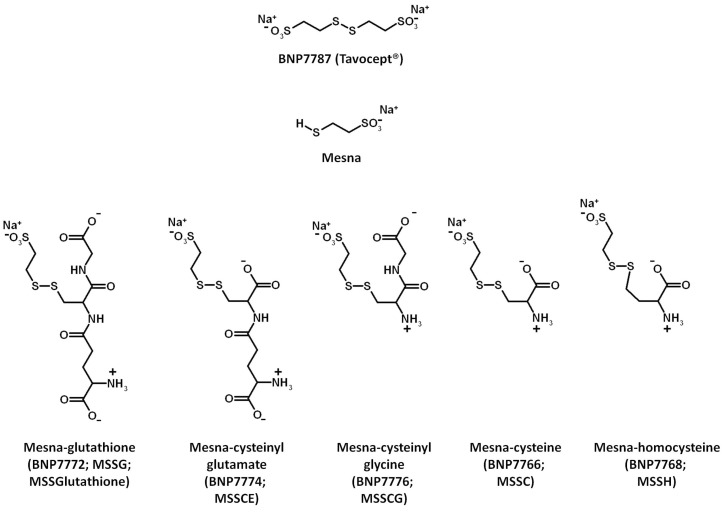
Structures of BNP7787, Mesna, and BNP7787-derived mesna-disulfide heteroconjugates.

Thioredoxin (Trx), glutaredoxin (Grx), and peroxiredoxin (Prx) are redoxin proteins that are important in regulating a variety of cell redox-related processes. Trx, Grx, and Prx have been implicated in a range of diseases including cancer and cardiovascular disease [1]. BNP7787 (Tavocept, Disodium-2,2'-dithio-bis-ethanesulfonate; Figure 1) is a novel, water soluble disulfide that has been evaluated in oncology-related clinical studies, and interaction of BNP7787 with different redoxin proteins has been a focus of research in our laboratory [2]. BNP7787 and BNP7787-derived mesna disulfide heteroconjugates act as alternative substrates for the Trx/Trx reductase coupled system, and BNP7787 targets specific cysteines on the redoxin protein Trx, resulting in reversible modulation of Trx activity [2]. We hypothesized that BNP7787 might promote formation of mixed disulfides on other functionally distinct redoxin proteins, like Grx and Prx, modulating protein function and/or activity.

Grx is a member of the glutaredoxin system along with glutathione reductase and glutathione peroxidase [3]. The glutaredoxin system is central to the transformations of glutathione, which is the dominant cellular thiol; Grx utilizes glutathione to reduce disulfides in cellular proteins, a process that yields glutathione disulfide as a by-product [4]. In addition to participating in the redox conversions of glutathione, when Trx is limiting, Grx can serve as a hydrogen donor for a number of cellular proteins, including ribonucleotide reductase, glutathione peroxidase, and methionine sulfoxide reductase [5,6]. There are at least four isotypes of Grx, and each isotype appears to have a distinct physiological function [7].

Prx is a member of the thioredoxin system that also includes the selenoprotein, thioredoxin reductase (Trx reductase) and Trx [8]. There are at least six isotypes of Prx in humans, and these Prx isotypes have important roles in intracellular redox balance and also in disease processes, including lung cancer [9,10,11]. Peroxiredoxins can be divided into three “types” or “classes:” typical 2-Cys (Prx 1-4), atypical 2-Cys (Prx 5), and 1-Cys peroxiredoxins (Prx 6). Peroxiredoxins catalyze reactions that are very different from the reactions catalyzed by Trx and Grx. Prx proteins convert hydrogen peroxide to water or alkyl peroxides to their corresponding alcohols. Trx is thought to act as a reductant in the second step of the Prx mechanism for both the typical and atypical 2-Cys Prx proteins. Prx proteins are also known as thioredoxin peroxidases or alkyl-hydroperoxide-reductases (for an excellent review of Prx proteins, please see Wood *et al.*) [12].

Grx and Prx expression and function have been correlated with a range of human diseases, including lung cancer, and mediate their effect on cell survival via unique intracellular pathways. Grx is overexpressed in human lung cancer tumor samples [13], Prx 1 is elevated in both adenocarcinoma and squamous cell lung cancer [14], and Prx 4 (Prx IV) is elevated specifically in adenocarcinoma non-small cell lung cancer (NSCLC) [9]. Additionally, interactions between Prx 4 and the redoxin sulfiredoxin, may influence lung cancer progression and/or metastasis [10]. 

BNP7787 is a multi-targeted investigational agent that we believe has the potential, *in vivo*, to simultaneously modify and modulate the function of a range of functionally diverse redoxin proteins. BNP7787 is generally well-tolerated and has been administered routinely at 18.4 g/m^2^, with observed C_max_ values of 10 mM and higher [15,16]. Herein, structure function data including activity assays, mass spectrometry characterizations, and X-ray crystallographic studies, elucidating interactions between BNP7787 and human Grx 1, human Prx 1, and human Prx 4 are presented. These data characterize covalent BNP7787-derived mesna moieties on cysteine residues of human Grx 1, Prx 1, and Prx 4 and provide information about the functional consequences of BNP7787-mediated modifications. Furthermore, the data presented herein combined with recently reported work on Trx [2], support our hypothesis that BNP7787 can interact with and modulate activity of the functionally distinct redoxin proteins: Trx, Grx and Prx *in vitro*. We propose that BNP7787 has the potential to affect the functioning of these different redoxin proteins *in vivo* as well. An oncology agent like BNP7787, with the potential to modulate the activity of proteins important in mediating different cell survival pathways, could have many potentially useful therapeutic applications.

## 2. Results and Discussion

As discussed above, Grx and Prx are functionally distinct redoxin protein families and we hypothesized that BNP7787 could modulate both Grx and Prx by targeting select cysteine residues and impacting function in a manner that is specific for each protein. To test this hypothesis, Grx 1, Prx 1 and Prx4 were selected as the focus of the studies described herein, and these selections were thought to be representative of the Grx redoxin family and the Prx redoxin family. Interactions between BNP7787 and Grx 1, Prx 1, and Prx 4 were simultaneously evaluated in a series of structure function related experiments that utilized *in vitro* activity assay methods, as well as mass spectrometry and X-ray crystallographic approaches, Section 2.1 and Section 2.2 below detail the *in vitro* assay and crystallographic data obtained for the interactions between BNP787 and Grx 1. Section 2.3 to Section 2.6 provide the *in vitro*, mass-spectrometry and X-ray crystallographic data detailing the interactions between Prx 1 and Prx 4 with BNP7787.

### 2.1. BNP7787 and BNP7787-Derived Mesna Disulfide Heteroconjugates are Alternative Substrates of the Redoxin Protein Grx 1

The effect of BNP7787 and BNP7787-derived mesna disulfide heteroconjugates on Grx 1 (hereafter Grx) was evaluated using three Grx-related reactions where NADPH oxidation was monitored (Table 1). Reactions included combinations of Grx, glutathione reductase, glutathione disulfide, BNP7787 and BNP7787-derived mesna disulfide heteroconjugates. The rate of Reaction 1 in Table 1, in the presence of Grx and GSH, gives the overall consumption by the Grx/glutathione reductase coupled system of BNP7787, BNP7787-derived mesna disulfide heteroconjugate, or any heteroconjugates formed with glutathione (GSH) in the reaction. In Reaction 1 containing Grx and GSH, Grx reduces BNP7787, BNP7787-related disulfide heteroconjugate, or any heteroconjugates formed with GSH. In this process Grx is oxidized, and oxidized Grx is reduced by GR, GSH and NADPH (NADPH provides the reducing equivalents). Reaction 1, in the presence of GSH but not Grx, monitors the glutathione reductase mediated reduction of BNP7787, BNP7787-derived mesna disulfide heteroconjugates, or heteroconjugates formed with GSH in the absence of Grx. In Reaction 1 where GSH is included but Grx is not, GSH may undergo thiol-disulfide exchange with the disulfides shown in Table 1, and the resulting mixed disulfides may be reduced by glutathione reductase. The rate of the reaction for a given disulfide, under conditions where neither Grx nor GSH is included, indicates the NADPH dependent reduction catalyzed by glutathione reductase alone.

For BNP7787 and the BNP7787-derived mesna disulfide heteroconjugates, turnover occurs most efficiently under the assay condition where Grx and GSH are both present (Table 1). The NADPH oxidation rates, for the reaction condition where Grx is not present, are notably lower for all of the compounds, reflecting the role that Grx has in optimal turnover of these compounds. Finally, when NADPH-mediated reduction of the compounds by glutathione reductase is examined in Reaction 1 in the absence of both Grx and glutathione, mesna-glutathione is a substrate but for the other compounds there is only negligible turnover. This might be expected given that oxidized glutathione is a substrate/cofactor of glutathione reductase. Cumulatively, the data in Table 1 indicate that BNP7787 and the BNP7787-derived mesna disulfide heteroconjugates act as alternative substrates for Grx when Grx is coupled to glutathione reductase. 

**Table 1:** The Interaction of BNP7787 and BNP7787-derived mesna disulfide heteroconjugates with glutaredoxin (GRX) (using glutathione reductase as a coupling enzyme with varying reaction conditions +/− Grx and/or +/− GSH).

	Reaction 1:		
	Glutathione reductase		
	X-S-X-Y + NADPH + H+ 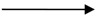 NADP+ + XSH + YSH		
Disulfide substrate (0.5 mM) *^a^*	Reaction 1 + Grx + GSH	Reaction 1 + GSH only	Reaction 1, no Grx, no GSH
	NADPH Oxidation (nmoles/min/mL) *^b,c^*		
BNP7787	15.3 ± 1.0	2.9 ± 1.6	0.0 ± 0.01
Mesna-glutathione	71.0 ± 7.9	11.3 ± 0.8	8.0 ± 0.6
Mesna-cysteine	28.3 ± 2.0	4.1 ± 1.3	0.0 ± 0.01
Mesna-homocysteine	10.7 ± 0.7	0.88 ± 0.2	0.16 ± 0.96
Mesna-cysteinyl glutamate	37.0 ± 2.1	2.4 ± 0.7	0.04 ± 0.12
Mesna-cysteinyl glycine	22.0 ± 0.5	4.1 ± 1.0	0.0 ± 0.07

*^a^* Disulfide structures are shown in Figure 1. *^b^* NADPH oxidation rates represent the average of rates from at least two independent experiments performed in triplicate (n = 6). *^c^* ANOVA analyses were performed on the whole dataset and for each disulfide. A two-way ANOVA analysis of the whole dataset indicates that the rate differences between all three reactions are significantly different among the disulfides tested (*p*-value = 0.001). One-way ANOVA analyses performed for each disulfide substrate indicate that (1) oxidation rates in the presence of Grx and GSH are significantly increased for each disulfide tested, and (2) the oxidation rates in the presence of GSH but not Grx were significantly increased for all the disulfides tested except mesna-glutathione and mesna-homocysteine.

These results of the *in vitro* assay above confirmed that BNP7787 and the related BNP7787-derived mesna disulfide heteroconjugates are alternative substrates for Grx. Studies to elucidate the interactions of BNP7787 and Grx, at the molecular level, are described below. BNP7787 is a disulfide and, as such, is capable of undergoing thiol-disulfide exchange with proteinaceous cysteine residues on Grx. Therefore, in addition to serving as an alternative substrate that can be turned over by Grx, BNP7787 may target and covalently modify select cysteine residues on Grx. 

### 2.2. BNP7787 Covalently Modifies Grx 1 on Cys7 and Cys82

**Figure 2:**
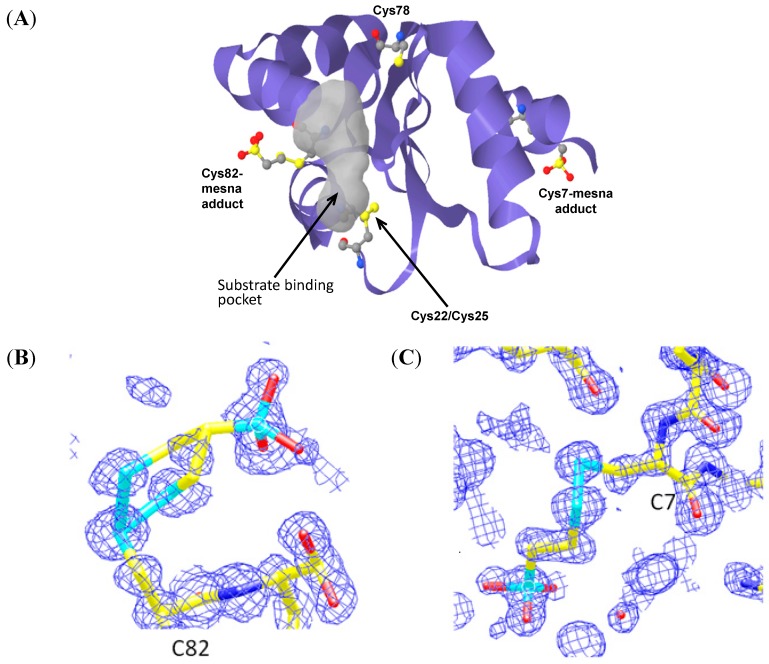
Structure of Grx 1-BNP7787 co-crystal. (**A**) Ribbon diagram of Grx 1 in complex with two BNP7787-derived mesna adducts at Cys 7 and Cys 82. (**B**) Atomic resolution map of BNP7787-derived mesna adduct formation on Cys 82. The adduct on Cys 82 adopts two conformations for S-C_β_ bond of the adduct whereas the adduct on Cys 7 is in one conformation. (**C**) Atomic resolution map of BNP7787-derived mesna adduct formation on Cys 7; the BNP7787-derived mesna adduct at Cys 7, the adduct is solvent exposed and does not have interactions with the protein (C = cysteine).

The elucidation of the molecular interactions occurring between BNP7787 and Grx were supported by the results of the Grx 1 related assays described above. A high resolution crystal structure was solved showing the atomic interactions between Grx 1 and BNP7787. The Grx-BNP7787 co-crystal crystallizes as a monomer in the asymmetric unit (Figure 2). BNP7787-derived mesna moieties were observed as mixed disulfides on Cys 7 and Cys 82. Both cysteines that interact with BNP7787 (Cys 7 and Cys 82) are solvent accessible. A close-up of the BNP7787-derived mesna adducts on Cys 7 and Cys 82 is shown in Figure 2 (Panels B and C). The BNP7787-derived mesna adducts on Grx 1 are located at a crystal contact and within close proximity of each other (data not shown).

### 2.3. BNP7787 Interactions with Redoxin Proteins Prx 1 and Prx 4

As discussed above, Grx and Prx are functionally distinct redoxin protein families and we hypothesized that BNP7787 could modulate both Grx and Prx by targeting cysteine residues and impacting function in a manner that is specific for each protein. To test this hypothesis, we also pursued studies on Human Prx 1 and Prx 4. Prx 1 and Prx 4 share high sequence identity with each other, and both of these Prx isotypes have been implicated in several types of lung cancer. Prx 1 is elevated in both adenocarcinoma and squamous cell lung cancer [14], while Prx 4 has been reported to be specifically upregulated in adenocarcinoma NSCLC [9]. Patients with advanced adenocarcinoma NSCLC receiving BNP7787 in combination with standard chemotherapy, have been observed to have substantial increases in overall survival [15]. To examine BNP7787’s role in modulating Prx, we pursued studies directed at identifying interactions that occur between BNP7787 and both Prx 1 and Prx 4 with results presented below in Section 2.4 through Section 2.6.

### 2.4. BNP7787 Covalently Modifies Prx 1 on Cys 52 and Cys 173 and Inhibits Prx 1 Activity

**Figure 3:**
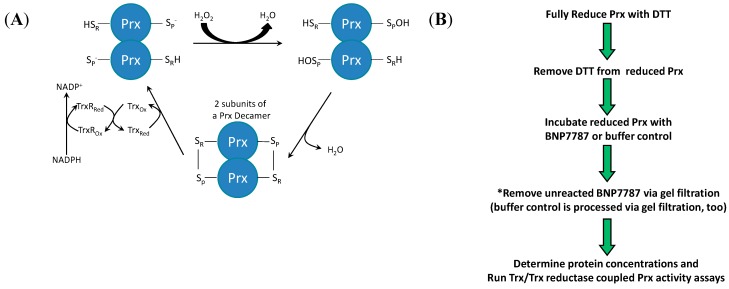

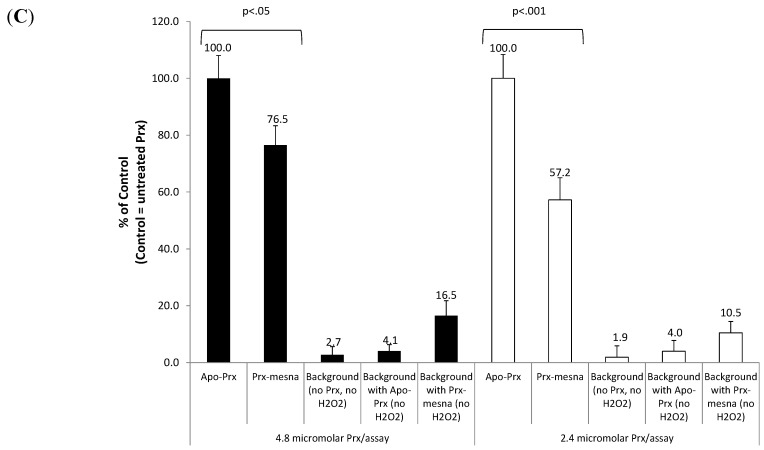
Effect of BNP7787 on Prx activity *in vitro*. (**A**) Prx enzyme assay (note that the assay is coupled to Trx/Trx reductase (TrxR)). Each Prx monomer contains an S_P_ and S_R_ cysteine; a Prx dimer is shown in this illustration. S_P_ refers to the cysteine that becomes oxidized to a cysteine sulfenic acid (peroxidatic cysteine) and S_R_ refers to the cysteine that attacks this cysteine sulfenic acid (resolving cysteine), forming a disulfide bond (this applies to typical 2-cys Prx and atypical 2-cys Prx species); (**B**) Preparation of BNP7787-modified Prx for activity assays (*Failure to remove unreacted BNP7787 will interfere with the Trx/Trx reductase coupled assay); (**C**) BNP7787 inhibits Prx activity in the Prx-Trx/Trx reductase coupled enzyme assay.

Peroxiredoxin (Prx) reduces hydrogen peroxide to water and alky peroxides to their corresponding alkyl alcohols [12]. Prx activity is typically monitored *in vitro* using a Trx/Trx reductase coupled assay (Figure 3A). We have previously reported that BNP7787 is an alternative substrate inhibitor of Trx [2]; therefore, to use the Trx-coupled Prx assay to evaluate the effect of BNP7787 on Prx activity unreacted/free BNP7787 must be removed prior to assaying Prx (Figure 3B and methods). If unreacted, free BNP7787 is not removed, the background NADPH oxidation rate of free BNP7787 masks the Prx reaction.

When Prx 1 is incubated with BNP7787, a putative Prx-mesna species is formed and the rate of Prx activity is notably reduced relative to apo-Prx control reaction rate. In assays containing 2.4 or 4.8 µM protein, the difference in relative rate of reaction between BNP7787-modified Prx (Prx-mesna) and apo-Prx was 43% and 23%, respectively (Figure 3C). The more pronounced effect at the lower Prx concentration of 2.4 µM is likely due to the fact that as levels of Prx increase, there is a higher proportion of unmodified Prx present in the assay in the Prx 1 samples incubated with BNP7787; therefore, as the proportion of unmodified Prx increases in a reaction, the turnover more nearly approaches the buffer control apo-Prx samples.

### 2.5. Identification of BNP7787-Derived Adducts on Cys 52 and Cys 173 of Prx 1 by Mass Spectrometry 

**Figure 4:**
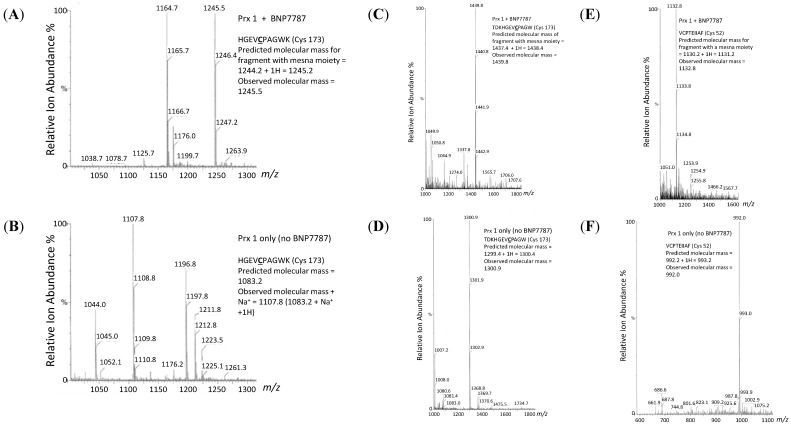
Representative positive ion ES mass spectrometry data for Prx 1 incubated with or without BNP7787. (**A**) Tryptic fragment of Prx 1 incubated with BNP7787 showing ion at *m/z* 1245.5 corresponding to fragment [HGEV**C**PAGWK + H]+ with BNP7787-derived adduct on Cys 173; (**B**) Tryptic fragment of Prx 1 incubated without BNP7787 did not have ion at *m/z* 1245.5 and was not modified on Cys 173; (**C**) Chymotryptic fragment TDKHGEV**C**PAGW of Prx 1 incubated with BNP7787 also showing BNP7787-derived adduct on Cys 173 of peroxiredoxin; (**D**) Chymotryptic fragment of Prx 1 incubated without BNP7787 was not modified on Cys 173 of Prx 1; (**E**) Chymotryptic fragment V**C**PTEIIAF of Prx 1 incubated with BNP7787 showing BNP7787 derived adduct on Cys 52 of Prx 1; (**F**) Chymotryptic fragment of Prx 1 incubated without BNP7787 had no adduct on Cys 52 of Prx 1.

To provide additional information about how BNP7787 was modulating Prx 1 function in the *in vitro* activity assays (Figure 3C) mass spectrometry analyses of the molecular interactions occurring between BNP7787 and Prx 1 were conducted. Adduct formation between Prx 1 and BNP7787 was analyzed using a combination of protein digests and mass spectrometry studies to identify the location of the putative adduct within the protein. A summary of the fragments generated from the trypsin and chymotrypsin digests that contain cysteine residues is shown in Table 2. Mass spectrometry data summarized in Figure 4 identified BNP7787-derived mesna adducts on Cys 52 and Cys 173 of Prx 1.

In these experiments, Prx was incubated with either BNP7787 or buffer, excess unreacted BNP7787 was removed from the Prx-BNP7787 reaction. Both the Prx-BNP7787 and the Prx-control (buffer only) samples were digested with either trypsin or chymotrypsin to provide fragments for LC MS analyses (Experimental Section 3.4). Mass spectrometry analyses of reactions using trypsin to fragment Prx1 revealed one BNP7787-derived mesna moiety on the Prx fragment HGEVCPAGWK (containing Cys 173) from Prx 1 incubated with BNP7787 (see Figure 4A). The *m/z* for this peak was 1245.5. In contrast, the Prx control reaction, where Prx was incubated only with buffer, did not have this ion and exhibited no peaks corresponding to this *m/z* (Figure 4B). The tryptic fragment containing Cys 52 was beyond the calibration window for the mass spectrometer in our laboratory; therefore, smaller fragments of Prx 1 containing Cys 52 were generated by chymotrypsin digestion. 

Mass spectrometry analyses of reactions using chymotrypsin to fragment Prx 1 confirmed the presence of one BNP7787-derived adduct on Cys 173 of peroxiredoxin in fragment TDKHGEVCPAGW with a *m/z* of 1439.8 (Figure 4C) and, therefore, was in agreement with the trypsin data described above. Prx 1 incubated without BNP7787 had no peaks corresponding to the *m/z* for the BNP7787-modified chymotryptic fragment TDKHGEVCPAGW (Figure 4D). Additionally, analysis of the chymotryptic fragmentation reactions of Prx 1 incubated with BNP7787 also identified a BNP7787 derived adduct on Cys 52 of Prx (Figure 4E). The *m/z* of this fragment, VCPTEIIAF; was 1132.8. This ion was absent in chymotryptic fragments of Prx 1 incubated without BNP7787 (Figure 4F). 

**Table 2:** Summary of tryptic and chymotryptic Prx 1 fragments containing cysteine residues analyzed by mass spectrometry for the presence of BNP7787-derived mesna adduct(s). (Note: the mass spectrometer utilized in these experiments was not calibrated to detect fragments larger than 2800 molecular mass. Therefore, experiments were done using both trypsin and chymotrypsin in order to generate digested fragments that contained all of the cysteine resides in peptides within the detection limit of the mass spectrometer).

Position of Cleavage	Resulting Peptide Sequence	Peptide Length [aa]	Peptide Mass [Da]	BNP7787-Derived Mesna Adduct Detected by Mass Spectrometry?
**Tryptic fragments containing cysteine residues**				
62	YVVFFFYPLDFTFV**C**PTEIIAFSDR(Cys 52)	25	^a^ 3037.5	^a^ This tryptic fragment was outside of the detection limit; but in a chymotrypsin digest (see below), modification of Cys 52 by BNP7787 was detected (see also Figure 4E)
92	LN**C**QVIGASVDSHF**C**HLAWVNTPK(Cys 71 & Cys 83)	24	2640.0	No
178	HGEV**C**PAGWK (Cys 173)	10	1083.2	Yes
**Chymotryptic fragments containing cysteine residues**				
59	V**C**PTEIIAF (Cys 52)	9	992.2	Yes
82	KKLN**C**QVIGASVDSHF (Cys 71) **or** N**C**QVIGASVDSHF (Cys 71)	16	1746.0 **or** 1376.5	No
87	**C**HLAW (Cys 83)	5	628.7	No
177	TDKHGEV**C**PAGW (Cys 173)	12	1299.4	Yes

^a^ Outside of the detection limit of the mass spectrometer used in these studies.

### 2.6. Identification of BNP7787-Derived Adducts on Cys 124 of Prx 4 by X-ray Crystallography 

**Figure 5:**
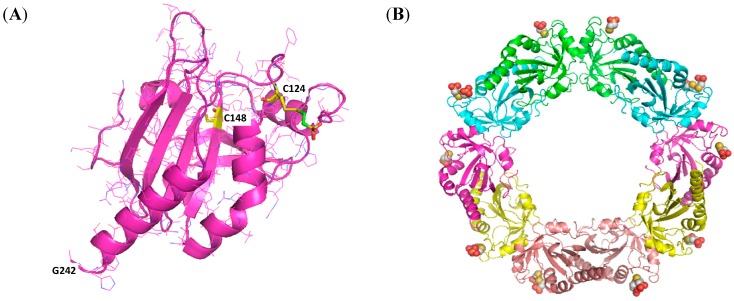
Ammonium sulfate condition crystals (1.6 M ammonium sulfate, 0.1 M MES pH 6.5, 10% dioxane) and the resultant high resolution Prx 4 crystal structure. (**A**) Structure of Prx 4 complexed with BNP7787-derived mesna moiety showing adduct formation at Cys 124 (C124) but not at Cys 148 (C148). Cys 245 (C245) was not visible in the electron density map; (**B**) The Prx 4 decamer generated by crystal symmetry.

As discussed in Section 2.4, mass spectrometry data indicated that Prx 1 was specifically modified on cysteines 52 and 173 of Prx 1. Because Prx 1 and Prx 4 have high sequence similarity, and because data indicate that Prx 4 has been specifically associated with adenocarcinoma of the lung [14], we pursued X-ray crystallography experiments with human Prx 4, simultaneously with the Prx 1 work described above, to unequivocally elucidate the location of BNP7787-derived modifications of cysteine residues on Prx 4 (Cys 52 in Prx 1 corresponds to Cys 124 in Prx 4, and Cys 173 in Prx 1 corresponds to Cys 245 in Prx 4). In X-ray crystallography experiments, a BNP7787-derived mesna adduct was detected on Cys 124 of Prx 4 (Figure 5). This modification of Cys 124 of Prx 4 corresponds to the modified Cys 52 on Prx 1. Although no BNP7787-derived adduct on Cys 245 of Prx 4 was detected by X-ray structure analyses (Cys 245 was not visible in the electron density map), mass spectrometry data are consistent with the idea that Cys 245 of Prx 4, which corresponds to Cys 173 of Prx 1, is also modified by BNP7787 as discussed in Section 2.6.2. 

#### 2.6.1. BNP7787 Prx 4 Co-Crystal: Protein Assembly and Domain Structure

**Figure 6:**
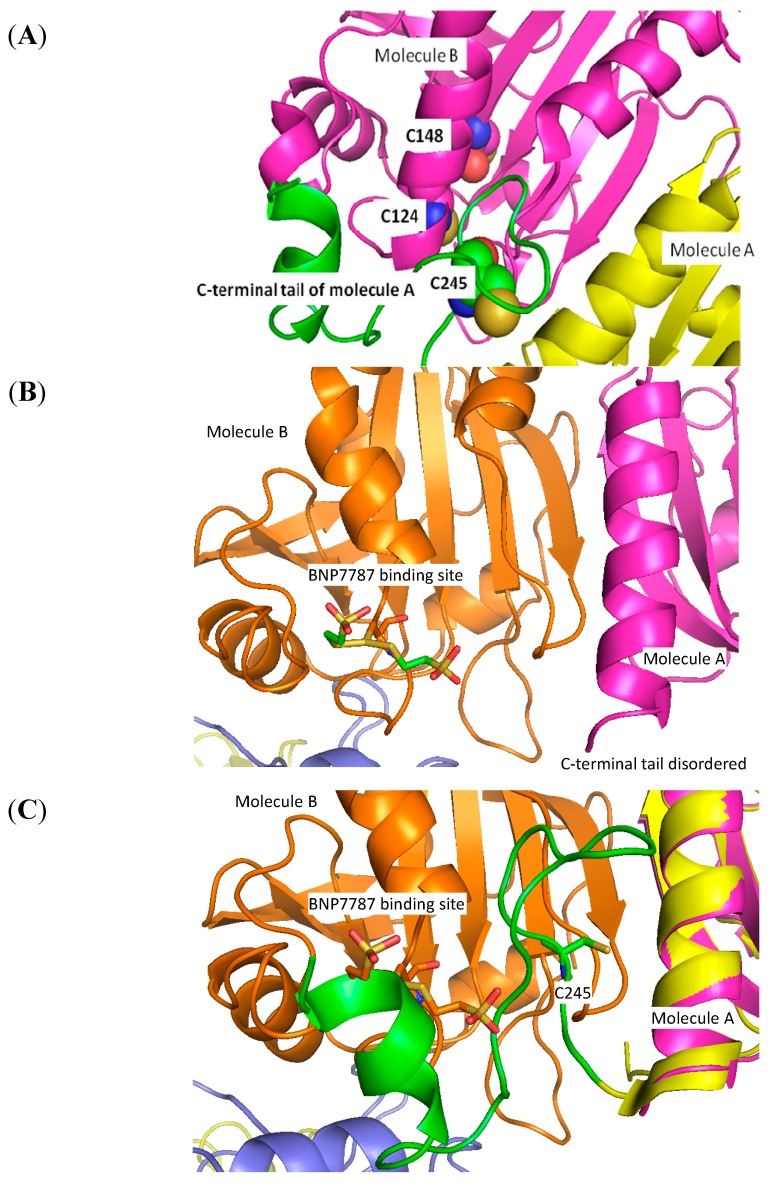

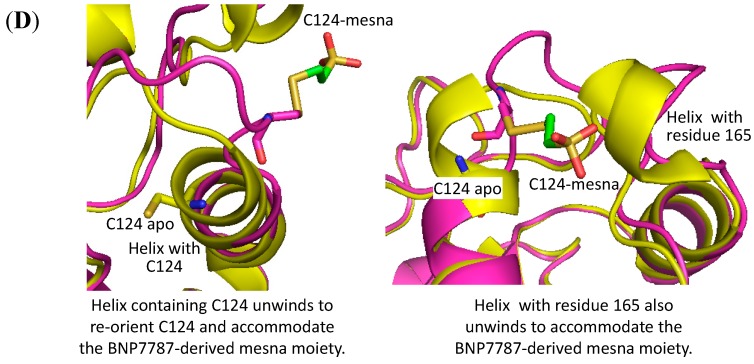
Ribbon diagrams of the Prx 4 crystal structure and the BNP7787-derived modifications of Prx 4. (**A**) Apo-Prx 4 structure showing multimer interface. The C-terminal tail (green) of molecule A (yellow) wraps around molecule B (pink) such that Cys 124 (C124) of molecule B is in close proximity to Cys 245 (C245) of molecule A; (**B**) Crystal structure of BNP7787-derived mesna adduct on Prx 4 showing BNP7787-derived mesna binding at Cys 124 and the C-terminal tail of molecule A ending at residue 242; (**C**) Overlay of the apo Prx 4 (Figure 6A) and the Prx 4 co-crystal containing a BNP7787-derived mesna (Figure 6B) structures showing a steric clash between the C-terminal tail of molecule A (apo, green/pink) and the BNP7787-derived mesna bound to molecule B (orange). (**D**) Close-up of BNP7787-derived mesna binding site showing partial unwinding of helix containing Cys 124 and unwinding of helix containing residue 165 to accommodate the BNP7787-derived mesna moiety at Cys 124 (apo Prx 4 is shown in yellow and the Prx 4-BNP7787 co-crystal is shown in pink).

**Figure 7:**
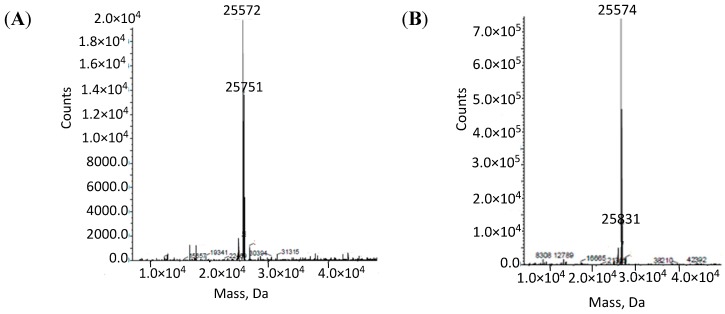
Mass Spectra (positive TOF) (**A**) Prx 4 after reaction with BNP7787, peak at 25572 corresponds to Prx monomer containing two BNP7787-derived mesna adducts (apo-protein is approximately 25292); (**B**) BNP7787-Prx 4 co-crystal protein sample after crystallization; crystals were dissolved and subjected to mass spectrometry analyses to confirm C-terminal tail was intact. The observed peak at 25574 corresponds well to the initially observed peak at 25572 suggesting no C-terminal proteolysis occurred during crystallization. (Note: low abundance peaks in the baseline are labeled with smaller font size.)

Apo-Prx 4 forms a donut shaped decamer. In the native apo structure (PDB ID: 2PN8), a C-terminal tail starting at Gly 242 wraps around a neighboring molecule forming an extended interface. At this interface, Cys 124 from one molecule is in close proximity to Cys 245 located on the C-terminal tail of an adjacent molecule (Figure 6A). BNP7787-modified Prx 4 also forms a donut shaped decamer (Figure 5B). As mentioned above, BNP7787 forms a mesna-adduct on Cys 124 (Figure 6B,C). Cys 148 is buried, well ordered, and does not appear to interact with the BNP7787-derived mesna moiety. The structure beyond residue 242, containing Cys 245 is disordered; therefore, from the X-ray crystal structure it is not possible to determine if Cys 245 is modified by BNP7787 or not. However, in Section 2.6.2, whole protein mass spectrometry data are presented suggesting that Prx 4 contains a second BNP7787-derived mesna adduct (Figure 7). Cys 124 and Cys 245 are catalytically important, conserved, active site residues. The BNP7787-derived mesna moiety was found to form a mixed disulfide with Cys 124 in all molecules of the decamer. In some molecules, the BNP7787-derived mesna moiety is bound in multiple conformations. To accommodate the BNP7787-derived mesna moiety, it appears that residues 121-126 undergo a conformational change partially unwinding the helix and exposing Cys 124 (Figure 6D). The presence of a BNP7787-derived mesna adduct with Cys 124 of Prx 4 would interfere with the placement of the section of structure containing Phe 267 (F267) from a neighboring molecule in the assembly (Figure 6C,D).

#### 2.6.2. Lack of Electron Density for the C-Terminal Region of the BNP7787-Prx 4 Co-Crystal

The absence of electron density beyond residue 242 of Prx 4 in the BNP7787-Prx 4 co-crystal structure is most likely due to a lack of a defined secondary or tertiary structure. As the binding of a BNP7787-derived mesna moiety would sterically interfere with the docking of the C-terminus portion of another Prx 4 molecule (as observed in the native apo structure), it is very likely that this segment is no longer composed of defined structural elements which would permit visualization in an electron density map. Specifically, Tyr 266 and Phe 267 of the native Prx 4 structure form a hydrophobic patch on the C-terminal helix and would normally occupy the same space as the BNP7787-derived mesna moiety and Cys 124. Disruption of this interaction could destabilize this helix further contributing to a lack of structure after residue 242. Since there was no electron density for the C-terminal tail, it may be in multiple and/or unstructured conformations

To evaluate if the lack of density for the C-terminal region was a result of limited proteolysis of the crystal reaction, mass spectrometry of the crystals was performed. The mass spectrum was consistent with intact protein (Figure 7) and suggested that the Prx 4 protein contained two BNP7787-derived mesna moieties after reaction with BNP7787. The mass of native protein is 25292 Da consistent with the N-terminal methionine residue being removed, and the mass of the modified protein prior to crystallographic analysis was 25572, consistent with two BNP7787-derived mesna adducts (Figure 7A). After crystallographic analysis mass was approximately 25574 (Figure 7B), also consistent with two BNP7787-derived mesna adducts. Based on the foregoing, lack of electron density in the C-terminal region of the X-ray structure of the BNP7787-Prx 4 co-crystal is probably not due to degradation of the C-terminal tail.

### 2.7. Mass Spectrometry and X-ray Crystallographic Studies Indicate that BNP7787-Mediated Modification of Grx and Prx Cysteine Residues is Selective with Target Specific Consequences

In the mass spectrometry experiments described above on Grx, BNP7787 modified Cys 7 and Cys 82 but not Cys 22, Cys 25 or Cys 78. On Prx 1, BNP7787 modified Cys 52 and Cys 173; however, no BNP7787-derived mesna adducts were identified on Cys 71 or Cys 83 of Prx 1. For studies of BNP7787 and Prx 4, Cys 124 was modified while Cys 148 was not modified. Data described herein, and the Trx-related data reported recently elsewhere [2], demonstrate that BNP7787 is able to specifically target cysteine residues for modification. Grx, Prx 1, and Prx 4 were incubated with concentrations of BNP7787 that would have facilitated modification of all cysteine residues, if BNP7787 interacted in a non-specific manner. However, the data herein indicate that BNP7787 interacts with and modifies specific cysteine residues on Grx, Prx 1, and Prx 4.

The modifications by BNP7787 have target specific consequences that are unique to each target with novel and distinct *in vitro* consequences that may translate to distinct, unique *in vivo* functional consequences. An area of ongoing and future research is related to elucidation of the molecular features required for BNP7787 to bind to and modify specific cysteine residues within proteins, but not other cysteine residues.

## 3. Experimental Section 

### 3.1. Materials and Methods

Peroxiredoxin 1 (Prx 1, human) and glutathione disulfide was purchased from SigmaAldrich (St. Louis, MO, USA). HPLC grade water and acetonitrile were obtained from Burdick and Jackson (VWR). Recombinant human glutaredoxin 1 (Grx 1) and bovine Trx reductase (used in experiments described in Table 1) were purchased from American Diagnostics, Inc. Human thioredoxin (Trx), glutathione, rat Trx reductase, and NADPH were purchased from SigmaAldrich (rat and human Trx reductase are more than 90% identical; rat Trx was used as a coupling enzyme for Prx assays). Ammonium bicarbonate (NH_4_HCO_3_) was purchased from Fluka. BNP7787 was prepared by a proprietary method (>97% purity, no mesna was detected by mass spectrometry). The BNP7787-derived mesna disulfide heteroconjugates (Figure 1) were prepared by a solid-state synthesis method [17] together with BioNumerik’s proprietary synthetic processes. Trypsin Gold was purchased from Promega; all other reagents were obtained from Sigma-Aldrich. Enzyme assays were carried out using a Molecular Devices SpectraMaxPlus UV/visible plate reader.

### 3.2. Coupled Glutaredoxin and Glutathione Reductase Assay

Grx and glutathione reductase activities with BNP7787 and BNP7787-derived mesna disulfide heteroconjugates were determined at 340 nm using the coupled assay described by Bjornberg and Holmgren [18]. In this assay, three types of reaction mixtures (Table 1) were prepared for each concentration of BNP7787 or BNP7787-derived mesna disulfide. All three reaction types contained 50 nM glutathione reductase and 400 μM NADPH in 0.10 M Tris-Cl, 2 mM EDTA, pH 8.0, and 100 μg/mL bovine serum albumin. One series of experiments additionally contained 0.025 μg of Grx and 1 mM GSH, and a second series of experiments additionally contained 1 mM GSH. BNP7787 or BNP7787-derived mesna disulfide heteroconjugates were added to reactions as 10x solutions in the assay buffer and final assay volume was 100 µL. Reactions were initiated by the addition of glutathione reductase and incubated for 10 min at room temperature. Activity was calculated using a linear portion from the first 4 min of each reaction. 

### 3.3. Trx/Trx Reductase Coupled Assay for Peroxiredoxin Activity 

Recently reported work from our laboratory has shown that BNP7787 is an alternative substrate for the Trx/Trx reductase coupled system [2]. Therefore, when BNP7787 is present in the Trx/Trx reductase assay, strong turnover of NADPH occurs as BNP7787 is reduced to mesna. Free BNP7787 will interfere with the Trx/Trx reductase coupled Prx assay resulting in high background turnover that masks the Prx turnover. Consequently, excess, unreacted BNP7787 must be removed from BNP7787-modified Prx in order to accurately assay BNP7787-modified Prx in the Trx/Trx reductase/Prx coupled assay system (Figure 3B). The steps for preparing and purifying the BNP7787-modified Prx 1 were as follows. Recombinant human Prx 1 (250 µg; 0.011 micromoles) was reduced using an excess of dithiothreitol (DTT; 83 mM final concentration) in NH_4_HCO_3_ buffer (40 mM, pH 8.0) at 37 °C for 1 h (total volume was 250 µM). DTT was removed using a NAP5 G25 Sephadex column (GE Life Sciences) and the DTT-free, reduced protein was incubated with BNP7787 (20 mM) or buffer alone at 37 °C (370 µL final volume) for 16 h. The BNP7787 and buffer only incubation reactions were chromatographed over G25 Sephadex columns. This step removed unreacted BNP7787 from the Prx 1 reaction incubated with BNP7787 and was used for the buffer control treated Prx 1 simply to ensure that both samples received the same handling/manipulation during the course of the experiment (final eluted volume was typically 850–1000 µL). The activity of apo-Prx 1 (Prx incubated with buffer) and Prx-mesna (BNP7787-treated Prx 1) was monitored using the coupled Prx assay (Trx and Trx reductase were coupling enzymes) that followed Prx 1-mediated NADPH oxidation at 340 nm [19]. A typical assay mixture contained HEPES Buffer (45 mM, pH 7.0, 1 mM EDTA), 350 µM NADPH, 0.12 µM rat liver Trx reductase, 80 µM insulin, 2.9 µM human Trx 1, and Prx 1 (4.8 or 2.4 µM per assay) and was 150 µL in final volume. Assays were initiated by the addition of H_2_O_2_ (100 µM) and were monitored at 340 nm at 25 °C for 30 min. Activity was calculated using a 4 min linear portion of each assay and the reaction rate was converted into % of control with the buffer treated Prx 1 reaction (apo-Prx) serving as a 100% control (Figure 3C). 

### 3.4. Preparation of BNP7787-Modified Prx 1 for Trypsin and Chymotrypsin Fragmentation and Subsequent Mass Spectrometry Analyses

Recombinant human Prx 1 (Sigma Aldrich; 333 µg; 0.015 micromoles) was reduced using an excess of dithiothreitol (DTT; 35 micromoles) in NH_4_HCO_3_ buffer (40 mM, pH 8.0) at 37 °C for 50 min (total volume was 750 µL; final Prx concentration was 20 µM and final DTT concentration was 46 mM). DTT was removed using a G25 Sephadex column (GE Life Sciences) and the DTT-free, reduced protein was incubated with BNP7787 (10 mM) or buffer alone at 30 °C (reactions 500 µL final volume) for 16–18 h (concentrations of BNP7787 of 10–15 mM have been observed in clinical studies and were well tolerated by patients [15,16]). Next, reactions were chromatographed over G25 Sephadex columns. This step removed unreacted BNP7787 from the Prx incubated with BNP7787 and was used for control (Prx incubated with buffer) to ensure that both samples received the same handling/manipulation during the course of the experiment (final eluted volume was typically 0.5–1 mL).

G25 chromatographed Prx1 incubation reactions were digested with Trypsin Gold or chymotrypsin. Trypsin Gold was dissolved in NH_4_HCO_3_ (400 µL) and acetonitrile (100 µL). This Trypsin Gold stock (100–150 µL) was added to the G25 chromatographed Prx 1 incubation reactions (500 µL). Next, acetonitrile (75 µL) was added and NH_4_HCO_3_ was added to a final volume of 750 µL. This reaction was incubated for 6 h at 37 °C. Chymotrypsin digests were prepared similarly. Chymotrypsin was dissolved in 1 M HCl and 50 µL was added to the G25 chromatographed Prx1 incubation reactions (500 µL) along with 10% v/v of CaCl_2_ (100 mM stock). Finally, NH_4_HCO_3_ buffer was added to give a final volume of 750 µL. Chymotrypsin digests were incubated for 16 h at 30 °C. Following digest by either trypsin or chymotrypsin, reactions were lyophilized to dryness and resuspended in a minimal amount of HPLC grade water in preparation for the liquid chromatography mass spectrometry analyses.

### 3.5. Liquid Chromatography Mass Spectrometry Analyses of Prx 1 Tryptic and Chymotryptic Fragments 

A Symmetry C18 HPLC column (Waters, Franklin, MA; 3.5 μm; 4.6 × 75 mm) and a Waters Alliance liquid chromatography system (Waters 2695, Franklin, MA, USA) coupled to a Micromass single quadropole mass detector (Micromass ZMD, Manchester, UK) were used to analyze fragments from trypsin or chymotrypsin digested human Prx1. The mobile phase contained 0.1% of formic acid throughout the run and the flow rate was 0.35 mL/min. The elution scheme involved the following steps: step 1: 0 to 3.5 min mobile phase was 95% water/5% acetonitrile; step 2: 3.5 to 20 min linear gradient to 10% water/90% acetonitrile; step 3: 20–30 min hold at 10% water/90% acetonitrile; step 4: 30–40 min linear gradient from 10% water/90% acetonitrile to 95% water; 5% acetonitrile. Positive-ion and negative-ion ionization modes across the mass ranges of 200–1200 Da (positive-ion mode) and 800–2800 Da (negative-ion mode) were used.

### 3.6. Cloning of Human Grx 1 and Prx 4

Wild-Type human glutaredoxin 1 (Grx 1) and human peroxiredoxin 4 (Prx 4) were cloned into a proprietary vector containing an N-terminal 6Xhis tag cleavable by TEV protease. Primers for Grx 1 were: 5' TATATA GGT ACC GCT CAA GAG TTT GTG AAC 3' and 5' TATATA GGA TCC TCA CTG CAG AGC TCC AA 3'. Primers for Prx 4 were: 5' TATATA GGT ACC GCG AAG ATT TCC AAG CC 3' and 5' TATATA CTC GAG TCA ATT CAG TTT ATC GAA AT 3'. Final products were sequence verified. 

### 3.7. Expression and Purification of Human Grx 1 and Prx 4

The final protein products for Grx 1 and Prx 4 were expressed in BL21(RIPL) cells. Cells containing the human Grx 1 and human Prx 4 constructs were grown at 37 °C to OD_600_ ~ 0.6. The cells were induced with 0.5 mM IPTG at 18 °C overnight. Cell biomass was harvested and stored at −80 °C. Purification of target protein was done in a 3 column system for Grx 1 and a 2 column system for Prx 4. The cell biomass was lysed by sonication in 50 mM Tris-HCl pH 7.8, 500 mM NaCl, 10% Glycerol, 20 mM Imidazole, 5 mM BME (Buffer A) plus 1 Roche Complete Protease Inhibitor Tablet, and 20,000 units Benzonase. Target protein was extracted by binding to Ni^2+^ charged IMAC resin and eluted using 250 mM imidazole for Grx 1 or a gradient of 0–500 mM imidazole for Prx 4. For Grx 1, peak fractions were cleaved with 3 mg TEV overnight in Buffer A. Cleaved protein was run over Ni^2+^ charged IMAC resin collecting the flow-through. Aggregated protein was separated from monomeric protein via size exclusion (S-75) in 50 mM Tris-HCl pH 7.5, 250 mM NaCl, and 5 mM DTT. Monomeric Grx 1 protein was concentrated to ~39 mg/mL. For Prx 4, peak fractions were pooled and aggregated Prx 4 protein was separated from monomeric protein via size exclusion in 10 mM HEPES pH 8.5, 300 mM NaCl, 5% glycerol, and 5 mM DTT. Monomeric Prx 4 protein was concentrated to approximately 12.6 mg/mL.

### 3.8. Preparation of BNP7787-Derived Mesna Adducts on Grx 1 and Prx 4

To prepare Grx 1 or Prx 4 that had been modified by BNP7787 for crystallization experiments, Grx 1 (39 mg/mL) was first incubated in 50 mM Tris, pH 7.5, 250 mM NaCl, and excess DTT (30–40 mM) or Prx 4 (12.6 mg/mL) was incubated in 10 mM Hepes, pH 8.5, 300 mM NaCl, 5% glycerol, and excess DTT (approximately 50 mM) for 1 h at 30 °C to fully reduce the protein, followed by incubation at 4 °C overnight. Excess DTT was removed from the Grx 1 reaction by exchanging the protein solution five times in 50 mM Tris, pH 7.5, and 250 mM NaCl, using ultrafiltration (10 kDa MWCO) and from the Prx 4 reaction by dialysis against 10 mM Hepes, pH 8.5, 300 mM NaCl, and 5% glycerol. Protein was supplemented with 1 mM DTPA, 1 mM neocuprione, and 40 mM BNP7787 and incubated at 4 °C overnight. Protein was screened by mass spectrometry to ensure that BNP7787-derived mesna adducts were detected (see above results) prior to crystallization attempts. A concentration of 40 mM BNP7787 was used in these crystallographic experiments to facilitate co-crystallization; however, BNP7787 is generally well tolerated by patients and doses of up to 18.4 g/m^2^, corresponding to 10–15 mM, have been routinely used in the clinic [15].

### 3.9. Crystallization of Grx 1 and Prx 4: Data Collection and Processing

To identify crystallization conditions, broad screens were set up using 96 well format (Greiner plates) at 20 mg/mL Grx 1 or 12.6 mg/mL Prx 4 with 1 mM DTPA, 1 mM neocuprione, 40 mM BNP7787 at 20 °C. The initial broad screens produced crystals under at least 4 conditions for both Grx 1 and Prx 4 and the screens used were PEGION, JCSG, BASIC, CRYSTALS HT, PEGRX, Pentaerythritol, Classics I , Classics II and/or PACT. Multiple rounds of fine screening were done at different protein concentration and different protein and reservoir ratios to obtain diffraction quality crystals. 

High resolution crystals were obtained for both Grx 1 and Prx 4. For Grx 1, co-crystals appeared in 4 conditions. The best Grx 1 crystals were grown in the presence of 20% PEG 8K, 0.1 M phosphate citrate pH 4.2, 0.2 M NaCl and diffracted to better than 1.1 Å. For Prx 4, co-crystals appeared in 2–3 conditions with the best crystals growing from 1.6 M ammonium sulfate, 0.1 M MES pH 6.5, 10% dioxane. Prx 4 crystals from the ammonium sulfate condition diffracted to 2.3 Å containing five molecules in the asymmetric unit (1/2 of the decamer donut biological unit). Prx 4 crystals were also obtained in 0.2 M ammonium phosphate, and diffracted to 2.95 Å (space group P21212) with 10 molecules (one decamer donut biological unit) in the asymmetric unit. This lower resolution structure agreed with the conformational changes observed in the high resolution structure ammonium sulfate Prx 4 adduct structure and also indicated the presence of one BNP7787-derived mesna adduct at Cys 124.

Prior to data collection, the crystals were transferred into a cryoprotectant solution made up of 25% ethylene glycol v/v for Grx, 35% glycerol v/v for Prx 4 in the ammonium sulfate crystallization buffer, and 40% glycerol v/v for Prx 4 in the ammonium phosphate crystallization buffer, after which they were flash-frozen in liquid nitrogen for data collection. Diffraction data for the Grx (1.1 Å) structure were collected at the Advanced Light Source (ALS) (Berkeley, CA, USA) on beamline 5.0.2 at an X-ray wavelength of 1.0 Å. BNP7787-derived mesna adducts were observed on Cys 7 and Cys 82 of Grx 1 and were clearly defined in the atomic resolution map. Data were processed using the program package MOSFLM as part of the CCP4 program package. Image processing statistics are summarized in Table 3. Diffraction data for the Prx 4 (2.3 Å) structure (ammonium sulfate crystallization condition) were collected at a wavelength of 1.0 Å on a Rayonix 300 detector array at beamline CLS-08ID at the Canadian Light Source, Saskatoon, Saskatchewan, Canada*.* A BNP7787-derived mesna adduct was observed on Cys 124 of Prx 4 (with a possible second adduct on Cys 245, see discussion in preceding sections). Imaging processing statistics for both Grx 1 and Prx 4 are summarized in Table 3.

**Table 3:** Grx 1 and Prx 4 crystal characteristics and data collection statistics (outer shell statistics shown in parentheses). (For Grx 1, note that low completeness in the outer shell was due to limits in detector geometry and not due to diffraction limits. All reflections were included in the refinement to provide the highest quality map. The overall completeness of the Grx 1 data set is 94% to 1.3 Å resolution).

	Grx 1	Prx 4
	PDB ID 4RQR	PDB ID 4RQX
*Data Processing*		
Unit cell (Å, °)	27.85, 55.41, 130.36, 90.00, 90.00, 90.00	108.00, 139.68, 96.19, 90.00, 103.17, 90.00
Space group	C222_1_	C2
Resolution range (Å)	27.71–1.08 (1.13–1.08)	50.00–2.27 (2.35–2.27)
No. of observations	165287	229144
No. of unique reflections	31162	62860
Redundancy	5.3 (2.5)	3.6 (3.0)
Completeness (%)	70.4 (13.2)	97.8 (92.1)
Mean I/σ (I)	19.5 (2.3)	13.0 (1.8)
R_merge_	0.051 (0.450)	0.075 (0.497)
*Refinement*		
Total no. of atoms	1077	6836
No. of protein molecules	1	5
No. of protein residues	107	830
No. of BNP7787-derived mesna adducts	2	5
Mean B-factor (Å^2^)	12.7	51.3
R_work_	0.172	0.208
R_free_	0.195	0.249
Rmsd bond lengths (Å^2^)	0.008	0.013
Rmsd bond lengths (°)	1.163	1.378
No. of disallowed φψ angles	0	0

### 3.10. Structure Solution and Refinement of the Grx 1 and Prx 4 Structures

Data for the Grx 1 (PDB ID: 4RQR) and Prx 4 structures (PDB ID: 4RQX) containing BNP7787-derived mesna adducts were indexed, integrated, scaled and merged using the programs HKL2000 or MOSFLM. The structures were solved by molecular replacement with *PHASER* using monomers from the Protein Data Bank entry for porcine Grx 1 (PDB ID: IKTE) and for human Prx 4 (PDB ID: 2PN8) as the search models. For Grx 1, the solution was consistent with one molecule in the crystal asymmetric unit. For Prx 4, the solution was consistent with five molecules in the crystal asymmetric unit. The protein models were iteratively refit and refined using *MIFit* and *REFMAC5* [20,21]. Final statistics for both Grx 1 and Prx 4 are summarized in Table 3.

The Grx 1 structure solution is supported by contiguous electron density for the entire chain trace of each molecule, landmark side chain density features matching the amino acid sequence including cysteines, absence of phi-psi violations and final R/Rfree values in the normal range. Residual density observed near Cys 7 and Cys 82 was modeled as BNP7787-derived mesna adducts. Residues Gln 39 and Glu 55 have missing side-chain atoms in the final structures (side chain atoms CD, CG, OE1, NE2 and CD, OE1, OE2, respectively).

For Prx 4, the segment from residues 121 to 126 containing Cys 124 was in a significantly altered conformation compared to the apo structure (PDB ID: 2PN8) and was consistent with the presence of a BNP7787-derived mesna adduct. The BNP7787-derived mesna adduct appears slightly disordered in the electron density map most likely due to its location on the surface of the protein. It is modeled in multiple conformations for some of the Prx 4 monomers (chains B, D, E). There is no density present beyond residue 242 (see results and discussion section). 

## 4. Conclusions

The redoxins Grx, Prx and Trx catalyze chemically distinct reactions and modulate different pathways involved in human disease processes. Grx is a small glutathione-dependent protein, which maintains a reduced state of cysteines in cellular proteins, and is primarily involved in catalyzing the reduction of glutathione mixed disulfides and producing glutathione disulfide as a by-product. In addition to participating in the redox conversions of glutathione, when Trx is limiting, Grx can also serve as a hydrogen donor for a number of cellular proteins, including ribonucleotide reductase, glutathione peroxidase, and methionine sulfoxide reductase [4,5,6]. Prx proteins are important in a variety of intracellular processes including modulation of effector molecules that regulate cell proliferation, cell differentiation and apoptosis; reduction of intracellular and biologically important peroxides including hydrogen peroxide and alkyperoxides; redox modulation of proteins important for transcription and cell proliferation [12].

The roles that Grx and Prx play in cancer involve different intracellular pathways that are unique to each protein. It has been reported that both Grx and Prx are overexpressed in several types of human lung cancer tumor samples [13,14]. Prx 4 is elevated specifically in the adenocarcinoma type of non-small cell lung cancer (NSCLC) [9], and it has been reported that interactions between Prx 4 and sulfiredoxin may influence lung cancer progression and/or metastasis [10]. BNP7787 is a novel, investigational agent that appears to have the potential to substantially increase patient survival in non-small cell lung cancer (NSCLC), especially in adenocarcinoma of the lung, the most common type of lung cancer [15]. As discussed in the introduction, BNP7787 has previously been administered at 18.4 g/m^2^, and concentrations of BNP7787 in the 10–15 mM range have been observed in the clinic. 

In experiments described herein, the molecular interactions between BNP7787 and the functionally distinct redoxin proteins, Grx and Prx, were evaluated using a combination of *in vitro* assays, mass spectrometry, and X-ray crystallography. These mass spectrometry and X-ray crystallography data unequivocally confirm that BNP7787 modifies Grx and Prx on select cysteine residues, and activity data indicate these interactions modulate protein function. Specifically, X-ray crystallography data herein shows that two of the five cysteines present in Grx 1 were modified, Cys 7 and Cys 82. Cys 22 and Cys 25 (active site residues) and Cys 78 were not modified. For Prx 1, mass spectrometry confirmed that 2 of the 4 cysteine residues (Cys 52 and 173) in Prx 1 were modified by BNP7787 while Cys 71 and 83 were not modified. For Prx 4, a BNP7787-derived mesna adduct was identified on Cys 124 using X-ray crystallography. The region of the Prx 4 crystal structure containing Cys 245 was disordered, and it was not possible to unequivocally determine that a BNP7787-derived mesna adduct was present. However, the whole protein mass spectrometry indicated that 2 molecules of BNP7787-derived mesna were present and, given that Cys 148 was not modified in the crystal structure, the data support a second BNP7787-derived mesna adduct on Cys 245. 

Tumor cells are genomically heterogeneous and contain subpopulations of cancer cells that often express different tumor-promoting proteins or that have multiple distinct dysregulated signaling pathways that modulate cell proliferation [22,23]. BNP7787 is an investigational small molecule disulfide that can modulate activity of proteins or protein families that are involved in regulation of distinct and separate cell pathways. This includes the redoxin proteins, Grx and Prx. Despite the fact that Grx and Prx catalyze very different reactions within the cell, simultaneously targeting the Grx family and the Prx family with a small molecule like BNP7787 could potentially be therapeutically advantageous and might be useful for modulating cell proliferation via distinct signaling pathways. BNP7787-mediated modifications occur on functionally important residues of Grx 1, and BNP7787 modifies Prx 1 and Prx 4 on catalytically important residues. Based on the new observations and experiments described herein, we propose that cysteine-specific modification, mediated by BNP7787, of the functionally distinct Grx and Prx redoxin proteins, results in selective modulation with the potential to impact cell growth and proliferation in a manner that is novel and specific for each protein target.
